# Risk factors for developing ventilator-associated lower respiratory tract infection in patients with severe COVID-19: a multinational, multicentre study, prospective, observational study

**DOI:** 10.1038/s41598-023-32265-5

**Published:** 2023-04-21

**Authors:** Luis Felipe Reyes, Alejandro Rodriguez, Yuli V. Fuentes, Sara Duque, Esteban García-Gallo, Alirio Bastidas, Cristian C. Serrano-Mayorga, Elsa D. Ibáñez-Prada, Gerard Moreno, Paula C. Ramirez-Valbuena, Gustavo Ospina-Tascon, Glenn Hernandez, Edwin Silva, Ana Maria Díaz, Manuel Jibaja, Magdalena Vera-Alarcon, Emili Díaz, María Bodí, Jordi Solé-Violán, Ricard Ferrer, Antonio Albaya-Moreno, Lorenzo Socias, William Figueroa, Jose L. Lozano-Villanueva, Fabio Varón-Vega, Ángel Estella, Ana Loza-Vazquez, Ruth Jorge-García, Isabel Sancho, Manu Shankar-Hari, Ignacio Martin-Loeches, LIVEN-Covid-19, Luis Antonio Gorordo, Ricardo Buitrago, Marcela Poveda, Lina Maria Saucedo, Elisa Estenssoro, Guillermo Ortiz, Nicolas Nin, Alfonso Jose Arango, Alvaro Aguilar, Andrea Lizeth Ayala, Andrea Viviana Bayona, Andrea Lizeth Ayala, Angelica Rodriguez, Carol Viviana Aponte, Carolina Forero-Carreño, Conny Stefanny Muñoz, Cristian Augusto Estrada, Cristopher Romero, Danilo Trujillo, Diego Holguin, Jesus Chavez-Villegas, Faure Rodriguez, Francisco Franco, Hernan Sánchez, Janett Vanessa Moncayo, Jennifer A. Pinedo, Jesica Valeria Bravo, Jose David Cruz, Jose Miguel Angel, Jovany Castro-Lara, Karen Andrea Mantilla, Lorena Garcia, Lorena Pabón, Luis Arturo Lopez, Luis Fernando Mamani, Marisa Lucrecia Yupa, Valeria Catalina Quevedo, SEMICYUC Study Group, Ana Loza, Diego Matallana Zapata, Isabel Díaz Torres, Sonia Ibañez Cuadros, María Recuerda Nuñez, Maria Luz Carmona Pérez, Jorge Gómez Ramos, Alba Villares Casas, María Luisa Cantón, José Javier González Contreras, Helena Pérez Chomón, Nerissa Alvarez Chicote, Alberto Sousa González, María De Alba Aparicio, Ruth Jorge García, Laura Sánchez Montori, Sandra Herrero García, Paula Abanses Moreno, Carlos Mayordomo García, Tomás Mallor Bonet, Paula Omedas Bonafonte, Enric Franquesa Gonzalez, Nestor Bueno Vidales, Paula Ocabo Buil, Carlos Serón Arbeloa, Isabel Sancho, Pablo Guerrero Ibañez, Pablo Gutierrez, María Concepción Valdovinos, Raquel Canto, Ruth Jorge García, Laura Sánchez Montori, Sandra Herrero García, Paula Abanses Moreno, Carlos Mayordomo García, Tomás Mallor Bonet, Paula Omedas Bonafonte, Enric Franquesa Gonzalez, Nestor Bueno Vidales, Paula Ocabo Buil, Carlos Serón Arbeloa, Isabel Sancho, Pablo Guerrero Ibañez, Pablo Gutierrez, María Concepción Valdovinos, Raquel Canto, Ana Luz Balán Mariño, María José Gutiérrez Fernández, Marta Martín Cuadrado, Belén García Arias, Lorena Forcelledo Espina, Lucía Viña Soria, Lorena Martín Iglesias, Lucía López Amor, Elisabet Fernández Rey, Emilio García Prieto, Débora Fernández Ruíz, Carla Martínez González, Lorenzo Socias, Marcio Borges‐Sá, María Aranda Pérez, Antonia Socias, José Ma Bonell Goytisolo, Inmaculada Alcalde Mayayo, Carlos Corradini, Isabel Ceniceros, Edwin Rodríguez, Jose Ignacio Ayestarán Rota, Mariana Andrea Novo, Joaquim Colomina Climent, Albert Figueras Castilla, Tomàs Leal Rullan, Maria Magdalena Garcias Sastre, Rossana Pérez Senoff, Ramón Fernández, Juan Carlos Martín González, Carmen Pérez Ortiz, José Luciano Cabrera Santana, Juan José Cáceres Agra, Domingo González Romero, Ana Casamitjana Ortega, Luis Alberto Ramos Gómez, Carolina Montelongo Ojeda, Jordi Solé-Violán, Alejandro Rodríguez, María Bodí, Gerard Moreno, Sandra Trefler, Laura Claverias, Raquel Carbonell, Erika Esteve, Montserrat Olona, Xavier Teixidó, Monserrat Vallverdú Vidal, Begoña Balsera Garrido, Elisabeth Papiol Gallofré, Raquel Albertos Martell, Rosa Alcaráz Peñarrocha, Xavier Nuvials Casals, Ricard Ferrer Roca, Eric Adrián Mayor Vázquez, Ferrán Roche Campo, Pablo Concha Martínez, Diego Franch Llasat, Joan Ramón Masclanz, Judith Marín‐ Corral, Purificación Pérez, Rosana Muñoz, Clara Vila, Francisco Javier González de Molina, Elisabeth Navas Moya, Josep Trenado, Imma Vallverdú, Eric Castañé, Emili Díaz Santos, Gemma Goma, Edg, Borja Suberviola, Antonio Albaya Moreno, Carlos Marian Crespo, Carmen Carolina Sena Pérez, Francisca Arbol Linde, Diana Monge Donaire, Vega Losada Martínez, Nuria Rodrigo Castroviejo, Gerardo Ferrigno, Reyes Beltrán, Carolina Sanmartino, Concepción Tarancón Maján, Alfredo Marcos Gutiérrez, Virginia Hidalgo Valverde, Caridad Martín López, Oihane Badallo, María del Valle Ortiz, Rebeca Vara Arlanzón, David Iglesias Posadilla, María Teresa Recio, Juan Carlos Ballesteros, Enrique Laza, Elena Gallego Curto, Ma Car‐men Sánchez García, Miguel Díaz‐Tavora, Rosa Mancha, Ana Ortega Montes, Isabel Gallego Barbachano, Eva Sanmartín Mantiñán, María Lourdes Cordero, Raquel María Rodríguez García, Jorge Gámez Zapata, María Gestal Vázquez, María José Castro Orjales, María Isabel Álvarez Diéguez, Carmen Rivero Velasco, Beatriz Lence Massa, María Gestal Vázquez, Ignacio Martí, Diego Matallana Zapata, Alberto Hernández Tejedor, Esther Ma López Ramos, Laura Alcázar Sánchez Elvira, Rocío Molina Montero, Ma Consuelo Pintado Delgado, María Trascasa Muñoz de la Peña, Yaiza Betania Ortiz de Zárate Ansotegui, Alejandra Acha Aranda, Juan Higuera Lucas, Juan Antonio Sanchez Giralt, Marta Chicot Llano, Nuria Arevalillo Fernández, Marta Sánchez Galindo, Ricardo Andino Ruiz, Alfonso Canabal Berlanga, Miguel Sánchez, Mercedes Nieto, Eduardo Arias Sarmiento, Adoración Bueno Blázquez, Rosa María de la Casa, Fátima Martín, Samuel González, Elena Martínez Quintana, Bernardo Gil Rueda, Áurea Higon Cañigral, Laura López Gómez, Pablo Safwat Bayoumi Delis, Augusto Montenegro Muore, Ángel Andrés Agamez Luengas, Enriqueta Andreu Soler, Ana Beatriz Pérez, José Higinio de Gea García, Rubén Jara Rubio, Silvia Sánchez Cámara, Alba Moreno Flores, José Moya Sánchez, Daniel Fran‐cisco Pérez Martínez, Ma Desamparados del Rey Carrión, María José Rico Lledó, Juana María Serrano Navarro, Juan Fran‐cisco Martín Ruíz, Julián Triviño Hidalgo, África López Ferrer, Isabel Cremades Navalón, Josefa Murcia Payá, J. M. Allegre Gallego, María del Carmen Lorente, Ruth González Natera, Raquel Garrido López de Murillo, Tania Ojuel Gros, Raquel Flecha Viguera, Isabel López González, Adriana García Herrera, Loreto Vidaur Tello, Maialen Aseguinolaza, Itziar Eguibar, Asunción Marqués Parra, Sergio García Marti, Alberto Lorenzo Aguilar, Laura Bellver Bosch, Victor Gascón Sanchez, Sonia De la Guía Ortega, Martín Parejo Montell, Alberto Belenguer Muncharaz, Hector Hernández Garces, Victor Ramírez Montero, Mónica Crespo Gómez, Verónica Martí Algarra, Susana Sancho Chinesta, Joaquin Arguedas Cervera, Faustino Álvarez Cebrian, Begoña Balerdi Pérez, Rosa Jannone Fores, Javier Botella de Maglia, Nieves Carbonell Monleón, Jose Ferreres Franco, Ainhoa Serrano Lazaro, Mar Juan Díaz, María Luisa Blasco Cortés, Laura Fayos, Julia Giménez, Gaspar Soriano, Ricardo Navarro, Sonia Mas, Elena Bisbal, Laura Albert, Johncard Romero, Juan Fernández Cabreara, Andrea Ortíz, Antonio Margarit Ribas, Neus Guasch

**Affiliations:** 1 Unisabana Center for Translational Science, Universidad de La Sabana, Chía, Colombia; 2 Clinica Universidad de La Sabana, Chía, Colombia; 3 Pandemic Sciences Institute, University of Oxford, Oxford, UK; 4 Critical Care Department, URV/IISPV/CIBERES, Hospital Universitari Joan XXIII, Tarragona, Spain; 5 Fundación Valle del Lili, Cali, Colombia; 6 Critical Care Department, Pontificia Universidad Católica de Chile, Santiago, Chile; 7 Fundación Clínica Shaio, Bogotá, Colombia; 8 Eugenio Espejo Hospital of Specialties, Quito, Pichincha Ecuador; 9 Critical Care Department, Hospital Universitari Parc Taulí, Universitat Autonoma Barcelona, Sabadell, Spain; 10 Hospital Universitario Dr Negrín, Las Palmas de Gran Canaria, Spain; 11 Universidad Fernando Pessoa, Canarias, Spain; 12 Vall d’Hebron Hospital Universitari, Barcelona, Spain; 13 Guadalajara University Hospital, Guadalajara, Spain; 14 Son Llatzer University Hospital, Palma de Mallorca, Spain; 15 Fundación Neumológica Colombiana, La Cardio, Bogotá, Colombia; 16 Jerez University Hospital, Jerez de la Frontera, Spain; 17 Critical Care Department, Hospital Universitario Virgen del Valme, Sevilla, Spain; 18 Hospital Providencial Nuestra Señora de Gracia, Zaragoza, Spain; 19 Critical Care Department, Hospital Universitario Miguel Servet, Zaragoza, Spain; 20 Intensive Care Unit, Royal Infirmary of Edinburgh, Little France Crescent, Edinburgh, UK; 21 Centre for Inflammation Research, The University of Edinburgh, Edinburgh, Scotland, UK; 22 Department of Intensive Care Medicine, Multidisciplinary Intensive Care Research Organization (MICRO), St. James’s Hospital, Dublin, UK; 23 Critical Care Unit, Hospital Juárez de México, Mexico, Mexico; 24 Hospital Interzonal de Agudos San Martín de La Plata, La Plata, Argentina; 25 Hospital Santa Clara, Bogota, Colombia; 26 Intensive Care Unit, Hospital Español, Montevideo, Uruguay; 27 UCI Hospital Universitario Virgen de Valme, Seville, Spain; 28 UCI Hospital Quirón, Huelva, Spain; 29 UCI Hospital Universitario Puerto Real, Cádiz, Spain; 30 UCI Hospital Universitario Virgen de la Macarena, Seville, Spain; 31 UCI Hospital Universitario Reina Sofía, Córdoba, Spain; 32 UCI Hospital Nuestra Señora de Gracia, Zaragoza, Spain; 33 UCI Hospital Clínico Universitario Lozano Blesa, Zaragoza, Spain; 34 UCI Hospital General San Jorge, Huesca, Spain; 35 UCI Hospital Obispo Polanco, Teruel, Spain; 36 UCI Hospital Universitario San Agustín, Avilés, Spain; 37 UCI Hospital Universitario Central de Asturias, Oviedo, Spain; 38 UCI Hospital Cabueñes, Gijón, Spain; 39 UCI Hospital Quirón Salud Palmaplanas, Palma de Mallorca, Spain; 40 UCI Hospital Universitario Son Espases, Palma de Mallorca, Spain; 41 UCI Hospital Comarcal d’Inca, Inca, Spain; 42 UCI Hospital Mateu Orfila, Mao, Spain; 43 UCI Complejo Hospitalario Universitario Insular—Materno Infantil, Las Palmas de Gran Canaria, Spain; 44 UCI Hospital General de la Palma, Tenerife, Spain; 45 UCI Hospital Universitari de Tarragona Joan XXIII, Tarragona, Spain; 46 UCI Hospital Universitari Arnau de Vilanova, Lleida, Spain; 47 UCI Hospital Verge de la Cinta, Tortosa, Spain; 48 UCI Hospital del Mar, Barcelona, Spain; 49 UCI Hospital Mutua de Terrasa, Terrasa, Spain; 50 UCI Hospital Sant Joan, Reus, Spain; 51 UCI Hospital Parc Tauli, Sabadell, Spain; 52 UCI Hospital Universitario Marqués de Valdecillas, Santander, Spain; 53 UCI Hospital Universitario de Guadalajara, Guadalajara, Spain; 54 UCI Hospital Nuestra Señora del Prado, Toledo, Spain; 55 UCI Hospital Virgen de la Concha, Zamora, Spain; 56 UCI Complejo Asistencial de Segovia, Segovia, Spain; 57 UCI Hospital universitario de Burgos, Burgos, Spain; 58 UCI Hospital Clínica de Salamanca, Salamanca, Spain; 59 UCI Hospital Universitario de Ceuta, Ceuta, Spain; 60 UCI Hospital San Pedro de Alcántara, Cáceres, Spain; 61 UCI Hospital de Mérida, Mérida, Spain; 62 UCI Hospital Montecelo, Pontevedra, Spain; 63 UCI CHUAC A Coruña, A Coruña, Spain; 64 UCI Centro Hospital Universitario de Ferrol, Ferrol, Spain; 65 UCI Hospitalario Clínico Universitario de Santiago, Santiago de Compostela, Spain; 66 REA CHUAC A Coruña, A Coruña, Spain; 67 UCI Hospital Lucus Augusti, Lugo, Spain; 68 UCI Hospital Infanta Elena, Huelva, Spain; 69 UCI IFEMA, Madrid, Spain; 70 UCI Hospital Príncipe de Asturias, Madrid, Spain; 71 UCI Hospital de la Princesa, Madrid, Spain; 72 UCI Hospital Clinico San Carlos, Madrid, Spain; 73 UCI Hospital HLA la Moncloa, Madrid, Spain; 74 UCI Hospital Morales Meseguer, Murcia, Spain; 75 UCI Hospital Clínico Universitario Virgen de la Arrixaca, Murcia, Spain; 76 UCI Hospital Reina Sofía, Murcia, Spain; 77 UCI Hospital Santa Lucía, Cartagena, Spain; 78 UCI Hospital Rafael Méndez, Lorca, Spain; 79 UCI Hospital Reina Sofía, Tudela, Spain; 80 UCI Hospital García Orcoyen, Estella‐Lizarra, Spain; 81 UCI Hospital Universitario de Donostia, Donostia, Spain; 82 UCI Hospital Universitario de La Ribera, Alzira, Spain; 83 UCI Hospital Dr. Peset, Valencia, Spain; 84 UCI Hospital Universitari i Politècnic La Fe, Valencia, Spain; 85 UCI Hospital Clínico Universitario de Valencia, Valencia, Spain; 86 UCI Hospital Virgen de los Lirios de Alcoy, Alicante, Spain; 87 UCI Hospital Arnau de Vilanova, Valencia, Spain; 88 UCI Hospital Comarcal de Vinarós, Vinarós, Spain; 89 ICU Hospital nostra Señyora de Meritxell, Les Esclades, Spain

**Keywords:** Virology, Medical research

## Abstract

Around one-third of patients diagnosed with COVID-19 develop a severe illness that requires admission to the Intensive Care Unit (ICU). In clinical practice, clinicians have learned that patients admitted to the ICU due to severe COVID-19 frequently develop ventilator-associated lower respiratory tract infections (VA-LRTI). This study aims to describe the clinical characteristics, the factors associated with VA-LRTI, and its impact on clinical outcomes in patients with severe COVID-19. This was a multicentre, observational cohort study conducted in ten countries in Latin America and Europe. We included patients with confirmed rtPCR for SARS-CoV-2 requiring ICU admission and endotracheal intubation. Only patients with a microbiological and clinical diagnosis of VA-LRTI were included. Multivariate Logistic regression analyses and Random Forest were conducted to determine the risk factors for VA-LRTI and its clinical impact in patients with severe COVID-19. In our study cohort of 3287 patients, VA-LRTI was diagnosed in 28.8% [948/3287]. The cumulative incidence of ventilator-associated pneumonia (VAP) was 18.6% [610/3287], followed by ventilator-associated tracheobronchitis (VAT) 10.3% [338/3287]. A total of 1252 bacteria species were isolated. The most frequently isolated pathogens were *Pseudomonas aeruginosa* (21.2% [266/1252]), followed by *Klebsiella pneumoniae* (19.1% [239/1252]) and *Staphylococcus aureus* (15.5% [194/1,252]). The factors independently associated with the development of VA-LRTI were prolonged stay under invasive mechanical ventilation, AKI during ICU stay, and the number of comorbidities. Regarding the clinical impact of VA-LRTI, patients with VAP had an increased risk of hospital mortality (OR [95% CI] of 1.81 [1.40–2.34]), while VAT was not associated with increased hospital mortality (OR [95% CI] of 1.34 [0.98–1.83]). VA-LRTI, often with difficult-to-treat bacteria, is frequent in patients admitted to the ICU due to severe COVID-19 and is associated with worse clinical outcomes, including higher mortality. Identifying risk factors for VA-LRTI might allow the early patient diagnosis to improve clinical outcomes.

**Trial registration:** This is a prospective observational study; therefore, no health care interventions were applied to participants, and trial registration is not applicable.

## Introduction

The Severe Respiratory Syndrome Coronavirus 2 (SARS-CoV-2) pandemic has affected over 500 million people worldwide, and almost 6 million people have died of this infection and its complications (https://covid19.who.int)^1,2^. SARS-CoV-2 causes coronavirus disease-19 (COVID-19), a multi-system illness with high morbidity and mortality related to progressive life-threatening pneumonia^3,4^. Patients frequently need ventilatory support due to the advanced disease severity^5–7^. Even though ventilatory supports are life-saving treatments, these are commonly associated with infectious and non-infectious complications^8–10^. Patients treated with ventilator support that develop secondary infections have more extended ICU and hospital length of stay, worsening clinical outcomes^11–15^.

Up to 50.5% of COVID-19 patients develop ventilator-associated lower respiratory tract infection (VA-LRTI), compared to 30.3% in patients with other severe pulmonary infections, such as influenza^16–18^. VA-LRTI is frequently categorised as ventilator-associated pneumonia (VAP) or ventilator-associated tracheobronchitis (VAT) in clinical practice and by international treatment guidelines^19^. Early reports highlighted that potentially difficult-to-treat bacteria (i.e., *Pseudomonas aeruginosa, Staphylococcus aureus, and Klebsiella pneumoniae,* among others) are common in VA-LRTI associated with COVID-19 in small studies mainly in high-income countries^20^. However, the underlying microbiology, the associated risk factors, and the impact on clinical outcomes for VA-LRTI in COVID-19 patients remain uncertain, especially in lower-income countries. Understanding risk factors related to VA-LRTI will suggest targeted prevention strategies, particularly within strained healthcare systems.

In this context, we conducted a multicentre, multinational, prospective observational cohort study in Latin America and Europe to characterise the cumulative incidence of VA-LRTI in COVID-19 patients, its underlying microbiology, associated risk factors, and its association with clinical outcomes.

## Materials and methods

This was a retrospective analysis of two prospective observational cohort studies of patients admitted to 84 ICUs due to severe SARS-CoV-2 infection in ten countries (i.e., Spain, Ireland, Andorra, Colombia, Chile, Ecuador, Mexico, Argentina, Uruguay, and Brazil) between March 2020 and January 2021. The patients were included in a voluntary registry created by the Latin American Intensive Care Network (Red LIVEN—https://www.redliven.org/web/)^21^ and the Spanish Society of Intensive Care Medicine (SEMICYUC)^21,22^. Data were collected prospectively by the attending physicians by reviewing medical records, laboratory data, and radiological records. All consecutive cases admitted who met the entry criteria were included in the registry. Both the Ethics Committee of the Clínica Universidad de La Sabana (IRB#2020AN28) and Hospital Joan XXIII (IRB#CEIM/066/2020) approved the study. All data were anonymized, allowing the requirement for informed consent to be waived. The ICU admission criteria and treatment decisions were left to the discretion of the attending physicians and were not provided in the study’s protocol.

The cohort includes patients older than 18 years hospitalised in the ICU due to severe SARS-CoV-2 infection and receiving invasive mechanical ventilation (IMV). Confirmed SARS-CoV-2 infection was determined by reverse transcription-polymerase chain reaction (rt-PCR) in a respiratory sample at each hospital based on local protocols. All COVID-19 patients admitted to the ICU with the clinical diagnosis of VAP or VAT were included in the VA-LRTI group. No patient received antibiotic treatment before the initiation of IMV. We excluded patients without pathogen isolated at the moment of the clinical diagnosis of interest (i.e., VAP or VAT). Patients that received IMV for less than 72 h were excluded. Finally, patients with a coinfection documented within the first 48 h of admission were excluded. All other patients were included and analysed in this study. The attending physician determined each patient's treatments and microbiological identification strategy.

The following variables were recorded during ICU admission: demographic data, comorbidities, symptoms, physiological variables collected during the first 24 h of ICU admission, systemic complications, and treatments used during the admission. For comparisons, patients admitted between February 22 and July 1, 2020, were classified in the subgroup of the “first wave,” while those admitted to the ICU until February 28, 2021 were considered a “second wave”. Variables with more than 30% missing data were discarded.

VA-LRTI was defined as a clinical syndrome of VAP or VAT in ventilated patients, as defined by the European Respiratory Society (ERS), European Society of Intensive Care Medicine (ESICM), European Society of Clinical Microbiology and Infectious Diseases (ESCMID), and Asociación Latinoamericana del Tórax (ALAT) guidelines^19^. VAP was defined as pneumonia that arises more than 48 h after endotracheal intubation; VAT as fever with no other recognizable cause, with new or increased sputum production, positive endotracheal aspirates (ETA) culture (> 106 CFU/mL) yielding a new bacterium and no radiographic evidence of nosocomial pneumonia. Additionally, only patients with the isolation of at least one respiratory pathogen known to be capable of causing pneumonia in a respiratory sample (e.g., tracheal aspirate, bronchoalveolar lavage, or pleural fluid) after the first 48 h of ICU admission were considered to have VA-LRTI.

The study aimed to determine the cumulative incidence, clinical characteristics, and outcomes of ventilated COVID-19 patients diagnosed with VA-LRTI. Moreover, to identify the risk factors associated with the development of VA-LRTI, the risk of hospital mortality among these patients, and describe the etiological microbiology.

### Statistical analysis

Categorical variables are presented in counts (percentages) and were evaluated through the Chi-square test. Continuous variables were expressed as median (interquartile ranges) or mean (standard deviation). T-Student test or Wilcoxon–Mann–Whitney test was performed according to data distribution.

A random forest (RF) model was used to predict the probability of hospital mortality. A total of 500 estimators were used in this model to calculate the area under the model’s receiver operating curve (AUROC), cross-validation was performed. A recursive elimination feature was carried out to select the smallest possible subset of variables that generate a model with adequate performance based on its AUROC. According to the Gini importance, the elimination is done by removing the variable with the least important from the dataset. To interpret the contribution of the optimal subset of variables in the model, the Python Treeinterpreter library was used. A multivariate logistic regression model was carried out with sociodemographic, admission data, and clinical outcomes variables identified as relevant in the final RF model (independent variables) to determine the Odds ratios (ORs) related to hospital mortality.

Another logistic regression model was developed to quantify the adjusted risk of VA-LRTI with all the sociodemographic, admission data, and clinical outcomes variables. A significance level of 0.05 and a confidence level of 95% were chosen. All statistical analysis was carried out in R studio 1.3.1056, Python 3.9.5–3.7.9, and IBM SPSS 28 for MAC. A more detailed description of the models and statistical analyses are presented in the online supplement.

## Results

**Figure 1:**
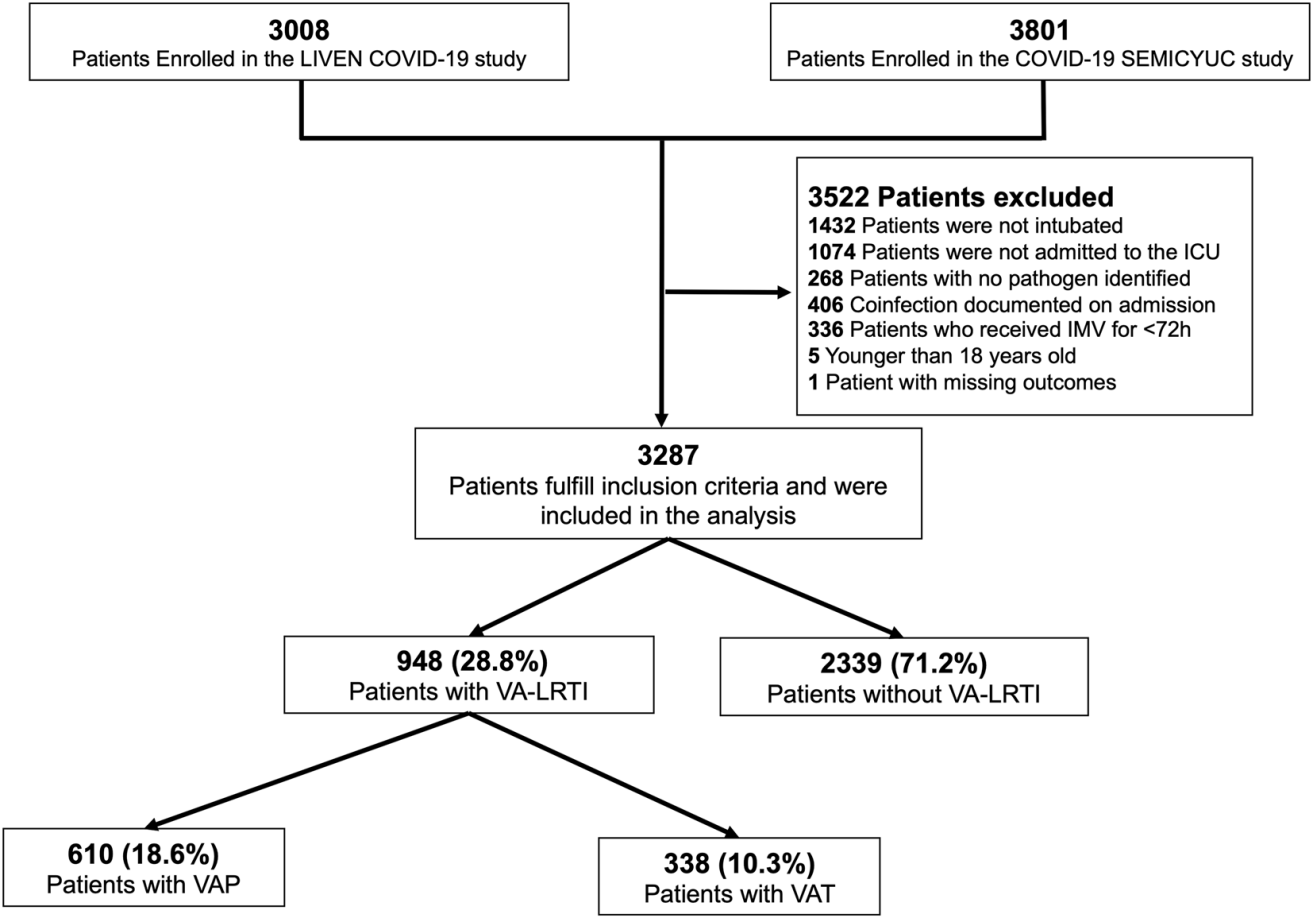
Flow chart of patients included in the analysis.

A total of 3287 patients were included in this study (Fig. 1, Table 1). The median [IQR] age was 63.0 years [54.5–71.0], and 70.5% [2317/3287] were male. Common comorbidities were hypertension 46.6% [1532/3287], obesity 36.4% [1193/3287], and diabetes mellitus 24.9% [819/3287].

**Table 1:** Characteristics and laboratory findings on the admission of the general cohort and VA-LRTI discriminated by patients that developed VAP and VAT.

	All cohort (n = 3287)	VAP (n = 610)	No VAP (n = 2677)	*p*-value	VAT (n = 338)	No VAT (n = 2949)	*p*-value
Age, median (IQR)	63.0 (54.5–71.0)	65.0 (57.0–72.0)	63.0 (54.0–71.0)	< 0.001	62.0 (54.0–69.8)	64.0 (55.0–71.0)	0.09
Male, n (%)	2317 (70.5)	442 (72.5)	1875 (70.0)	0.26	236 (69.8)	2081 (70.6)	0.83
Health worker, n (%)	79 (2.4)	12 (2.0)	67 (2.5)	0.53	9 (2.7)	70 (2.4)	0.89
Influenza vaccine, n (%)	59 (1.8)	1 (0.2)	58 (2.2)	0.001	29 (8.6)	30 (1.0)	< 0.001
First wave, n (%)	2516 (76.5)	371 (60.8)	2145 (80.1)	< 0.001	302 (89.4)	2214 (75.1)	< 0.001
Second wave, n (%)	771 (23.5)	239 (39.2)	532 (19.9)	< 0.001	36 (10.6)	735 (24.9)	< 0.001
Comorbid condition, n (%)							
Congestive heart failure	172 (5.2)	22 (3.6)	150 (5.6)	0.06	23 (6.8)	149 (5.1)	0.21
Hypertension	1532 (46.6)	311 (51.0)	1221 (45.6)	0.018	145 (42.9)	1387 (47.0)	0.17
COPD	250 (7.6)	51 (8.4)	199 (7.4)	0.49	22 (6.5)	228 (7.7)	0.49
Asthma	140 (4.3)	33 (5.4)	107 (4.0)	0.15	4(1.2)	136 (4.6)	0.004
Chronic kidney disease	185 (5.6)	26 (4.3)	159 (5.9)	0.13	30 (8.9)	155 (5.3)	0.009
Neurologic disease	47 (1.4)	7 (1.2)	40 (1.5)	0.64	7 (2.1)	40 (1.4)	0.42
Haematological disease	90 (2.7)	29 (4.8)	61 (2.3)	0.001	5 (1.5)	85 (2.9)	0.19
HIV-AIDS	10 (0.3)	2 (0.3)	8 (0.3)	0.77	4 (1.2)	6 (0.2)	0.010
Obesity	11,960 (36.4)	221 (36.2)	975 (36.4)	0.97	85 (25.2)	1111 (37.7)	< 0.001
Rheumatological disease	105 (3.2)	17 (2.8)	88 (3.3)	0.61	4 (1.2)	101 (3.4)	0.039
Diabetes	819 (24.9)	156 (25.6)	662 (24.7)	0.70	93 (27.5.3)	725 (24.6)	0.27
ARDS at admission	721 (2.9)	59 (9.7)	662 (24.7)	< 0.001	183 (54.1)	538 (18.2)	< 0.001
Number of comorbidities, median (IQR)	1.0 (0.0–2.0)	1.0 (1.0–2.0)	1.0 (0.0–2.0)	0.06	1.0 (0.0–2.0)	1.0 (0.0–2.0)	0.028
Laboratory testing, median (IQR)							
Creatinine	0.9 (0.7–1.2)	0.9 (0.7–1.2)	0.9 (0.7–1.2)	0.72	0.9 (0.7–1.2)	0.9 (0.7–1.2)	0.60
Leucocytes	9.3 (6.6–13.1)	9.4 (6.7–13.2)	9.3 (6.6–13.1)	0.66	11.1 (7.8–15.8)	9.1 (6.5–12.9)	< 0.001
CRP	16.7 (9.0–28.5)	15.6 (97.8–25.0)	17.0 (9.1–29.3)	< 0.001	27.2 (12.0–152.5)	16.0 (8.7–27.0)	< 0.001
Procalcitonin	0.3 (0.2–0.4)	0.3 (0.1–0.4)	0.3 (0.2–0.4)	0.003	0.3 (0.3–0.8)	0.3 (0.2–0.4)	< 0.001

*IQR* interquartile range, *COPD* chronic pulmonary injury, *HIV-AIDS*: human immunodeficiency virus-acquired immunodeficiency syndrome, *Leucocytes* leucocytes 109 cells/L, *C-RP* C-reactive protein mg/dL.

### Cumulative incidence of VA-LRTI by pandemic waves and by geographic region

A total of 28.8% [948/3287] patients were diagnosed with VA-LRTI. VAP cumulative incidence was 18.6% [610/3287], and VAT cumulative incidence was 10.3% [338/3287]. Most of the patients in our study were enrolled during the first pandemic wave (76.5% [2516/3287]), with a higher VAP cumulative incidence observed during the second wave (14.7% [371/2516] vs. 31.0% [239/771], *p* < 0.001). Also, VA-LRTI proportion was higher in Latin America than Europe (36.7% [368/1004] vs. 25.4% [580/2283], *p* < 0.001).

### Microbiology

**Figure 2:**
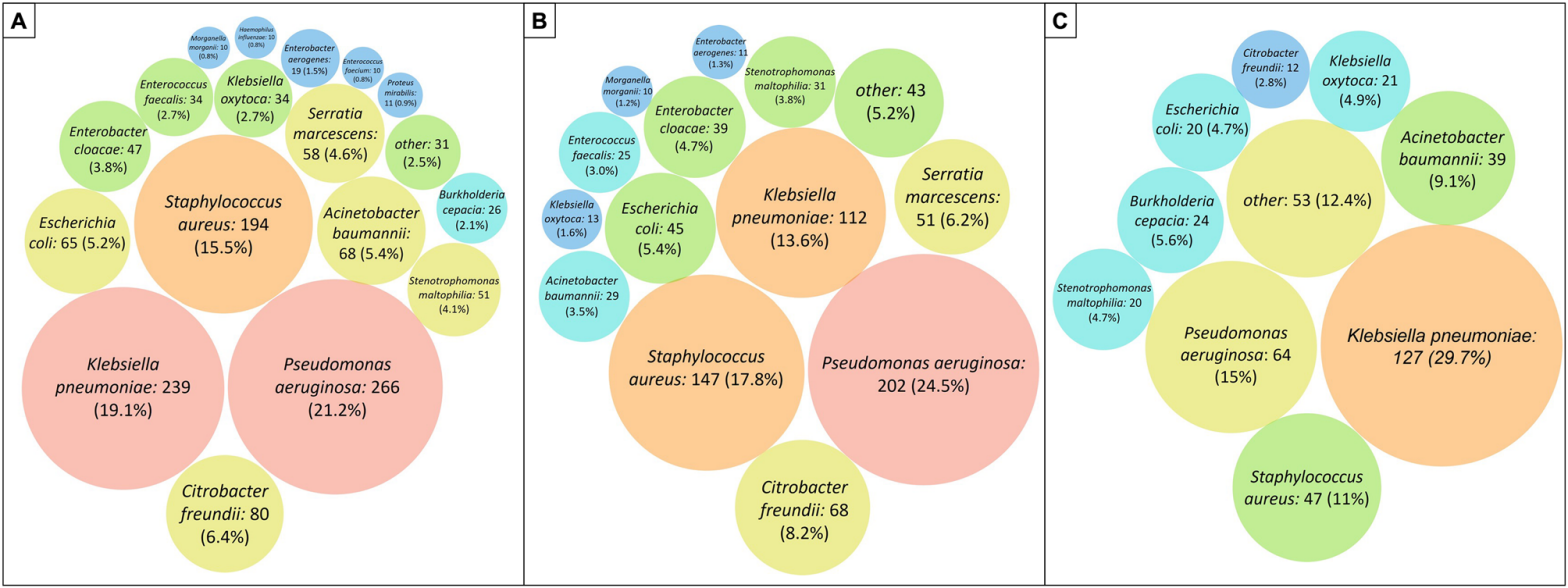
Aetiological pathogens. Here is presented the frequency (in counts) of the etiological isolations in the whole cohort of patients with VA-LRTI. (**A**) Shows microorganisms isolated in the general cohort. (**B**) Represents the VAP cohort and Panel C depicts the VAT cohort. Within the *“Other”* group are microorganisms with less than five isolates (*Achromobacter xylosoxidans*, *Acinetobacter lwoffii*, *Citrobacter koseri*, *Hafnia alvei*, *Moraxella catharralis*, *Neisseria cinerea*, *Providencia rettgeri*, *Raoultella planticola*, *Raoultella ornithinolytica*, *Streptococcus anginosus*, and *Streptococcus pyogenes*).

A total of 1252 bacteria species were isolated. The most frequently identified pathogens were *Pseudomonas aeruginosa* (21.2% [266/1253]), followed by *Klebsiella pneumoniae* (19.1% [239/1253]), *Staphylococcus aureus* (15.5% [194/1253]), and *Citrobacter freundii* (6.4% [80/1253]). For VAT, the most frequently identified pathogens were *K. pneumoniae* (29.7% [127/427]), followed by *P. aeruginosa* (15% [64/427]), and for VAP most frequent pathogens were *P. aeruginosa* (24.5% [202/826]) followed by *S. aureus* (17.8% [147/826]). Notably, most identified pathogens were Gram-negative roots and potentially difficult-to-treat bacteria (Fig. 2). We did not collect information on antibiotic resistance patterns in these pathogens.

Several differences were found between Latin America’s and Europe’s microbiology. Regarding the most frequently identified bacterial pathogens in Latin America were *Klebsiella pneumoniae* (33.5% [164/489), followed by *Pseudomonas aeruginosa* (12.5% [61/489]), and *Staphylococcus aureus* (10.6% [52/489). While in Europe, *Pseudomonas aeruginosa* (26.8% [205/764]), *Staphylococcus aureus* (18.6% [142/764]), and *Citrobacter freundii* (10.1% [77/764]) were the most frequently identified bacteria. Notably, *Acinetobacter baumannii* was Latin America's fourth most prevalent bacteria (10.6% [52/489]). In contrast, this bacteria was not as predominant in Europe (2.1% [16/764]) (Table 2).

**Table 2:** Identified microbiology in the general cohort and VA-LRTI discriminated by Latin American and European patients.

Microbiology identification			
Microrganism	All cohort n = 1253	Latin America n = 489	Europe n = 764
*Pseudomonas aeruginosa*	266 (21.2)	61 (12.5)	205 (26.8)
*Klebsiella pneumoniae*	239 (19.1)	164 (33.5)	142 (18.6)
*Staphylococcus aureus*	194 (15.5)	52 (10.6)	77 (10.1)
*Citrobacter freundii*	80 (6.4)	3 (0.6)	75 (9.8)
*Acinetobacter baumannii*	68 (5.4)	52 (10.6)	47 (6.2)
*Escherichia coli*	65 (5.2)	26 (5.3)	39 (5.1)
*Serratia marcescens*	58 (4.6)	11 (2.2)	36 (4.7)
*Stenotrophomonas maltophilia*	51 (4.1)	15 (3.1)	34 (4.5)
*Enterobacter cloacae*	47 (3.8)	13 (2.7)	28 (3.7)
*Enterococcus faecalis*	34 (2.7)	6 (1.2)	16 (2.1)
*Klebsiella oxytoca*	34 (2.7)	23 (4.7)	11 (1.4)
*Burkholderia cepacia*	26 (2.1)	24 (4.9)	8 (1.0)
*Enterobacter aerogenes*	19 (1.5)	11 (2.2)	8 (1.0)
*Proteus mirabilis*	11 (0.9)	6 (1.2)	7 (0.9)
*Enterococcus faecium*	10 (0.8)	3 (0.6)	7 (0.9)
*Morganella morganii*	10 (0.8)	2 (0.4)	5 (0.7)
*Haemophilus influenzae*	10 (0.8)	3 (0.6)	4 (0.5)
*Streptococcus pneumoniae*	7 (0.6)	4 (0.8)	3 (0.4)
*Achromobacter xylosoxidans*	4 (0.3)	0 (0.0)	3 (0.4)
*Acinetobacter lwoffii*	4 (0.3)	3 (0.6)	2 (0.3)
*Streptococcus pyogenes*	4 (0.3)	4 (0.8)	2 (0.3)
*Citrobacter koseri*	3 (0.2)	1 (0.2)	1 (0.1)
*Hafnia alvei*	3 (0.2)	0 (0.0)	1 (0.1)
*Moraxella catharralis*	1 (0.1)	0 (0.0)	1 (0.1)
*Neisseria cinerea*	1 (0.1)	0 (0.0)	1 (0.1)
*Providencia rettgeri*	1 (0.1)	0 (0.0)	1 (0.1)
*Raoultella planticola*	1 (0.1)	1 (0.2)	0 (0.0)
*Raoultella ornithinolytica*	1 (0.1)	1 (0.2)	0 (0.0)
*Streptococcus anginosus*	1 (0.1)	0 (0.0)	0 (0.0)

### VA-LRTI clinical characteristics and outcomes

Patients who developed VAP and VAT had similar age (median [IQR] VAP: 65.0 years [57.0–72.0]; VAT: 62.0 years [54.0–69.8]), and were most often male (VAP: 72.5% [442/610]; VAT: 69.8% [236/338]), and had similar laboratory results (Table 1). The most prevalent comorbidities were hypertension, obesity, and diabetes in both groups (Table 1).

Regarding treatments during hospital admission, those who developed VAP more often received corticosteroid treatment (VAP: 74.9% [457/610]; VAT: 70.4% [238/338]), while those with VAT more frequently received lopinavir/ritonavir (VAP: 1.9% [50/610]; VAT: 0.9% [25/338]). The antimicrobial usage was similar among groups. Patients who develop VAP had longer hospital LOS when compared to VAT (median [IQR] VAP: 40 days [27.0–57.0]; VAT: 24.5 days [16.0–44.0]) and more days under IMV (median [IQR] VAP: 25.9 days [16.0–38.0]; VAT: 15.0 days [11.0–25.0]). Finally, the prevalence of acute kidney injury (AKI) during ICU stay was similar among both groups (VAP: 42.0% [256/610]; VAT: 41.4% [140/338]) as well the mortality rates (VAP: 43.9% [268/610]; VAT: 47.3% [160/338]) (Table 3).

**Table 3:** Treatment, interventions, and outcomes of the general cohort and VA-LRTI discriminated by patients that developed VAP and VAT.

Treatments, interventions and clinical outcomes							
	All cohort (n = 3287)	VAP (n = 610)	No VAP (n = 2677)	*p*-value	VAT (n = 338)	No VAT (n = 2949)	*p*-value
ECMO, n (%)	92 (2.8)	21 (3.4)	71 (2.6)	0.35	7 (2.1)	85 (2.9)	0.49
Prone position, n (%)	2498 (76.0)	507 (83.1)	1991 (74.4)	< 0.001	277 (82.0)	2221 (75.3)	0.008
Corticosteroids use, n (%)	2413 (73.4)	460 (75.4)	1953 (73.0)	0.23	249 (73.7)	2164 (73.4)	0.96
One corticosteroid, n (%)	2363 (71.9)	457 (74.9)	1906 (71.2)	0.18	238 (70.4)	2125 (72.1)	< 0.001
Two corticosteroid, n (%)	92 (2.8)	16 (2.6)	76 (2.8)	0.18	50 (14.8)	42 (1.4)	< 0.001
Hydrocortisone, n (%)	200 (6.1)	36 (5.9)	164 (6.1)	0.91	66 (19.5)	134 (4.5)	< 0.001
Methylprednisolone, n (%)	1096 (31.4)	200 (32.8)	896(33.5)	0.78	98 (29.0)	998 (33.8)	0.08
Dexamethasone, n (%)	1251 (38.1)	2530 (41.5)	998 (37.3)	0.06	174 (51.5)	1077 (36.5)	< 0.001
Dexamethasone + hydrocortisone, n (%)	61 (1.9)	12 (2.0)	49 (1–8)	0.95	41 (12.1)	20 (0.7)	< 0.001
Dexamethasone + methylprednisolone, n (%)	22 (0.7)	1 (0.2)	21 (0.8)	0.16	9 (2.7)	13 (0.4)	< 0.001
Methylprednisolone + hydrocortisone, n (%)	9 (0.3)	3 (0.5)	6 (0.2)	0.48	0 (0.0)	9 (0.3)	0.64
Lopinavir/ritonavir, n (%)	51 (1.6)	50 (1.9)	1 (0.2)	< 0.01	25 (0.9)	26 (7.7)	< 0.001
Interferon beta, n (%)	1 (0.0)	1 (0.0)	0 (0.0)	0.42	1 (0.0)	0 (0.0)	0.19
Neuraminidase inhibitor, n (%)	58 (1.8)	58 (2.2)	0 (0.0)	< 0.001	57 (1.3)	1 (0.3)	0.05
Ampicilin/sulbactam, n (%)	5 (0.2)	5 (0.2)	0 (0.0)	0.62	5 (0.2)	0 (0.0)	0.98
Amoxacilin/clavulanate, n (%)	3 (0.1)	3 (0.1)	0 (0.0)	0.93	3 (0.1)	0 (0.0)	0.72
Ampicilin, n (%)	3 (0.1)	3 (0.1)	0 (0.0)	0.93	3 (0.1)	0 (0.0)	0.72
Azitromicine, n (%)	3 (0.1)	3 (0.1)	0 (0.0)	0.93	3 (0.1)	0 (0.0)	0.72
Claritromycine, n (%)	5 (0.2)	5 (0.2)	0 (0.0)	0.62	5 (0.2)	0 (0.0)	0.98
Amikacine, n (%)	6 (0.2)	6 (0.2)	0 (0.0)	0.52	1 (0.0)	5 (1.5)	< 0.001
Ceftriaxone, n (%)	1 (0.0)	1 (0.0)	0 (0.0)	0.42	1 (0.0)	0 (0.0)	0.19
Vancomicine, n (%)	1 (0.0)	1 (0.0)	0 (0.0)	0.42	1 (0.0)	0 (0.0)	0.19
Linezolid, n (%)	2 (0.1)	2 (0.1)	0 (0.0)	0.81	2 (0.1)	0 (0).0	0.49
Tocilizumab, n (%)	21 (0.6)	21 (0.8)	0 (0.0)	0.06	14 (0.5)	7 (2.1)	< 0.01
Rendesivir, n (%)	9 (0.3)	7 (0.3)	2 (0.3)	0.88	5 (0.2)	4 (1.2)	< 0.01
Hydroxychloroquine/chloroquine, n (%)	82 (2.5)	81 (3.0)	1 (0.2)	< 0.001	63 (2.1)	19 (5.6)	< 0.001
Outcomes							
AKI, n (%)	1147 (34.9)	256 (42.0)	891 (33.3)	< 0.001	140 (41.4)	1007 (34.2)	0.027
Deaths, n (%)	1285 (39.1)	268 (43.9)	1017 (38.0)	0.007	160 (47.3)	1125 (38.2)	0.001
Hospital LOS, median (IQR)	26.0 (17.0–42.5)	40.0 (27.0–57.0)	24.0 (15.0–18.0)	< 0.001	24.5 (16.0–44.0)	27.0 (17.0–42.0)	0.66
LOS, median (IQR)	17.0 (10.0–28.0)	29.0 (19.0–45.0)	15.0 (9.9 -23.0)	< 0.001	18.0 (12.0–28.0)	16.0 (10.0–28.0)	0.007
IMV Days, median (IQR)	14.0 (8.0–24.0)	25.0 (16.0–38.0)	12.0 (7.0–20.0)	< 0.001	15.0 (11.0–25.0)	14.0 (8.0–24.0)	0.004

### Impact of VA-LRTI on hospital mortality

**Figure 3:**
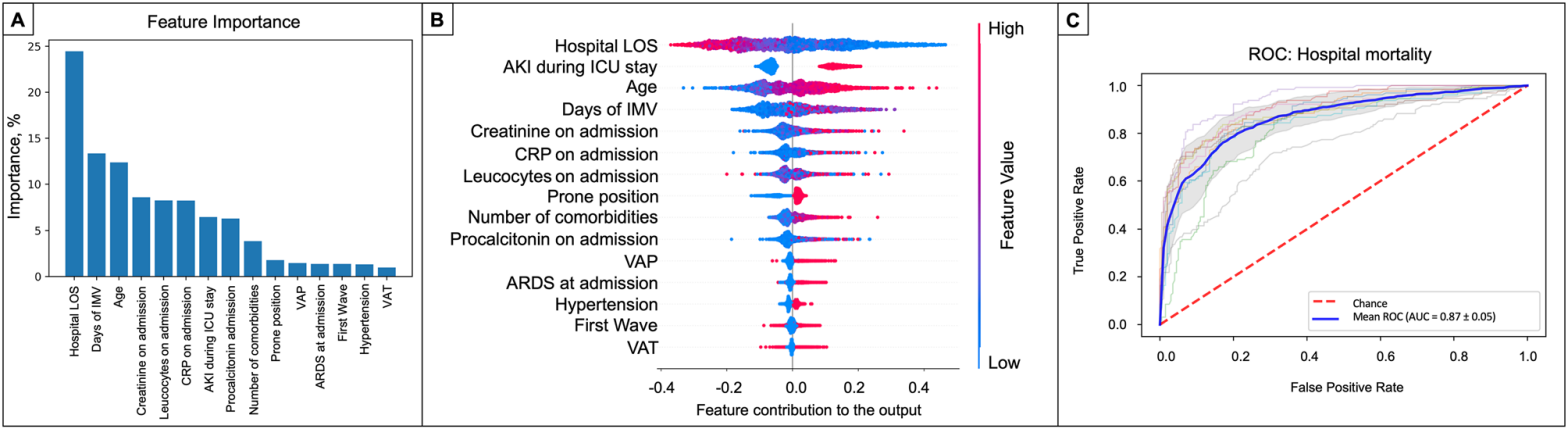
An automatized model to determine the impact of ventilator-associated lower respiratory tract infection on hospital mortality. (**A**) Presents variables more strongly associated with hospital mortality according to the Gini importance. (**B**) Represents the contribution of the variables to the output; the red values indicate a high-value contribution of the variable, and the blue values are a low-value contribution. The positive values in the plot indicate a high probability of hospital mortality, and negative values indicate a low likelihood of hospital mortality. (**C**) Presents each cross-validation trial’s receiver operative curve (ROC) for the subset of the selected variables. The blue curve represents the average of the ROC curves of each test, and the average area under de ROC is also presented. The most significant variables associated with hospital mortality according to their Gini importance and contribution to the output were Hospital-LOS, higher days under IMV, old age, higher creatinine levels on admission, and lower PaO2/FiO2 on ICU admission. Also, the development of AKI during ICU stays and VA-LRTI were risk factors associated with hospital mortality.

**Figure 4:**
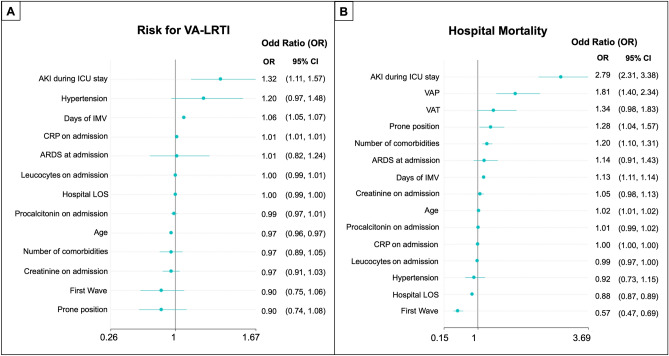
Logistic regression models to identify factors associated with ventilator-associated lower respiratory tract infection (VA-LRTI) (**A**) and the impact of VA-LRTI on hospital mortality (**B**). Logistic regression was performed with the optimal subset of variables obtained with the random forest model for the two outcomes (VA-LRTI and hospital mortality). The odds ratios (OR) are graphically represented in the Forest plot for better medical interpretability. (**A**) Presents the odd proportions of the risk for VA-LRTI, and (**B**) is shown the odds ratios for the hospital mortality.

The Random Forest (RF) analyses identified AKI, VAP development, prone position ventilation, number of comorbidities, and days under IMV as risk factors associated with higher hospital mortality. Immunomodulant and antiviral treatments showed less than 1% importance according to the Gini feature importance; thus, these were unrelated to the addressed outcome. The ROC curve of the constructed model using the selected variables in the RF had a mean [SD] of 0.87 [± 0.05] to predict hospital mortality (Fig. 3). Moreover, in the multivariate logistic regression analysis, we found an OR [95% CI] of 2.79 [2.31–3.38] for AKI during ICU stay and 1.81 [1.40–2.34] for VAP. However, the association between VAT and hospital mortality was not statistically significant, despite the higher point estimates (1.34 [0.98–1.83]) (Fig. 4).

### Risk factors associated with the development of VA-LRTI

In the RF analysis and the logistic regression analysis carried out to identify the variables associated with the development of VA-LRTI, we found that the development of AKI during ICU stay (OR, 95% CI 1.32 [1.11–1.57]), along with days under IMV (OR, 95% CL 1.06 [1.05–1.07]) and high CRP serum concentration on ICU admission (OR% CL: 1.01 [1.01–1.01]) were significantly associated with the development of this VA-LRTI (Fig. 4). Notably, prior history of Diabetes Mellitus, hypertension, and obesity were not associated with the development of VA-LRTI.

### Ethics approval and consent to participate

Both the Ethics Committee of the Clínica Universidad de La Sabana (IRB#2020AN28) and Hospital Joan XXIII (IRB#CEIM/066/2020) approved the study. All patients signed informed consent to participate in the study. Local guidelines and international regulations were taken into account, and the Declarations of Helsinki and Colombian Resolution No 008430 of 1993.

## Discussion

We found that VA-LRTI occurs in more than a quarter of patients with severe COVID-19 admitted to the ICU, often with potentially difficult-to-treat pathogens, and regional differences in incidence (higher incidence in Latin America). The most frequently isolated pathogen was *Pseudomonas aeruginosa*. AKI during ICU stay, longer duration under IMV, and higher CRP levels at ICU admission were risk factors independently associated with the development of VA-LRTI. Notably, severe COVID-19 patients who developed VAP had higher mortality, while the association between VAT and hospital mortality was not statistically significant.

Our study found a cumulative incidence of 28.8% for VA-LRTI in patients with severe COVID-19 that required IMV and admission to the ICU, which is consistent with the systematic reviews that highlight an incidence varying between 21 to 64%^23,24^. Pulmonary superinfections are reported to be more frequent in COVID-19 patients compared to other viral aetiologies^18,25,26^. Of note, the cumulative incidence of VAT is estimated to be 14% in the European population^18^. Importantly, we found that the cumulative incidence of VA-LRTI (i.e., VAP and VAT) was higher in patients enrolled in Latin America when compared to the incidence in European Countries during the same study period, which is a novel finding. To the best of our knowledge, these differences have not been described in the literature. Notably, these differences build into the argument that each country and hospital should have local protocols and guidelines; and that one size does not fit all when discussing infection prevention.

We found that the most frequent etiological microorganisms of VA-LRTI isolated in the respiratory samples were *Pseudomonas aeruginosa, Klebsiella pneumoniae*, and *Staphylococcus aureus,* which are considered potentially challenging to treat pathogens as they tend to either generate new or have resistance to carbapenems, and beta-lactams, amongst other antibiotics used in this context^27–31^. Although a similar prevalence of difficult-to-treat pathogens has been reported previously^18,32^, these were smaller cohorts, restricted to European countries. Moreover, the microbiology observed in Latin America and Europe was notably different. *Klebsiella pneumoniae* was the principal bacteria in Latin America. This data is concerning since this microorganism may become frequently resistant and constitutes a challenging pathogen to treat, especially in countries with limited resources and no new antibiotics being widely available. It is important to highlight that *Klebsiella pneumoniae* has been highly reported in patients with COVID-19 in Latin America^33,34^. Interestingly, the prevalence of *Acinetobacter baumannii* was particularly higher in Latin America, which is also concerning. Rangel et al. found one of the highest *Acinetobacter baumannii* resistance rates in Latin America. This infection is increasing in COVID-19 patients due to disease severity and has been associated with prolonged hospitalization and associated immune dysfunction^35^. Thus, our study adds to this key literature by highlighting geographic differences and including patients in limited-resource settings such as South America.

We found that patients who developed VAP but not VAT were independently associated with increased hospital mortality, which is consistent with previous studies^23,36^. Interestingly, the higher risk of mortality observed in patients ventilated in the prone position was unexpected, given the potential value of the prone position in severe ARDS. However, this study was not intended to explore the effect of this therapy on clinical outcomes. We hypothesise that the prone position might facilitate the dissemination of microorganisms to the lower respiratory tract^37–39^, leading to a greater risk for VAP development. Nonetheless, a 2022 systematic review and network meta-analysis including 20 randomised clinical trials state that the prone position did not increase the VAP incidence^40^. Thus, more studies are required to understand this finding further. Of note, factors associated with mortality were similar to those variables associated with VA-LTRI, such as AKI during admission, days under mechanical ventilation, and the number of comorbidities. Identifying risk factors for the development of VA-LRTIVAP is pivotal to optimizing preventive strategies and hypothesizing potential underlying mechanisms^25,41–43^.

Since the pandemic’s beginning, several studies have attempted to identify the factors associated with increased mortality in patients with severe COVID-19. The most frequent factors associated with worse clinical outcomes in patients with severe COVID-19 are older age, male gender, several comorbid conditions, dexamethasone treatment, the development of AKI during admission, and more days under mechanical ventilation^12,14,22,44–47^. For instance, a study conducted with 9657 patients showed an increased incidence of AKI in COVID-19 patients that was associated with a significant increase in the risk of death (HRs of 3.4 [95% CI 3.0–3.9] for AKI^25,41,42^. Since COVID-19 patients have a greater risk of AKI development^48^, it is critical to prevent this complication. In this regard, the 25th Acute Disease Quality Initiative (ADQI) Workgroup recommends healthcare professionals individualise fluid and haemodynamic management based on a dynamic assessment of the cardiovascular status and limit nephrotoxic drug exposure where possible^49^.

We report the largest cohort of COVID-19 patients that evaluate risk factors and mortality associated with VA-LRTI. Our cohort patients from very different health care settings in 10 countries, from Europe and Latin America, involving different COVID-19 waves, giving external validity. We adopted the international consensus case definitions for our exposure of interest (VA-LRTI), had positive culture to inform LRTI diagnoses, and used an outcome without ascertainment bias (hospital mortality). Although our study did not include standardized protocols to prevent and diagnose VA-LRTI, the participating centres used internationally accepted guidelines to prevent and diagnose VA-LRTI in patients with severe COVID-19, including pneumonia zero protocols. We did not collect time from intubation to the development of VA-LRTI or the resistance patterns of the identified pathogens, which is a limitation and should be taken into account for future research. However, we did include the number of days patients remained under IMV, which may serve as a surrogate. Finally, although our study did not include standardized protocols for the treatment of COVID-19 patients between the different ICUs, the WHO recommendations were broadly followed in the participating centres.

In conclusion, ventilator-associated lower respiratory tract infections in patients with COVID-19 are frequent and associated with higher mortality, especially in those that develop VAP. Studies aimed to identify the mechanisms responsible for these complications and trials of prevention strategies are needed to identify potential treatments that could improve outcomes.

## Supplementary Information

Supplementary Information.

## Data Availability

The datasets used and/or analysed during the current study are available from the corresponding author on reasonable request.

## Abbreviations

ICU: Intensive care unit
VA-LRTI: Ventilator-associated lower respiratory tract infection
COVID-19: Coronavirus disease-19
HIV/AIDS: Human immunodeficiency virus/acquired immunodeficiency syndrome
SARS-CoV-2: Severe Respiratory Syndrome Coronavirus 2
VAP: Ventilator-associated pneumonia
VAT: Ventilator-associated tracheobronchitis
rt-PCR: Reverse transcription-polymerase chain reaction
IMV: Invasive Mechanical Ventilation
ERS: European Respiratory Society
ESICM: European Society of Intensive Care Medicine
ESCMID: European Society of Clinical Microbiology and Infectious Diseases
ALAT: Asociación Latinoamericana del Tórax
ETA: Endotracheal aspirates
LOS: Length of stay
RF: Random forest
AUROC: The area under the model’s receiver operating curve
ORs: Odds ratios
IQR: Interquartile range
CRP: C reactive protein
AKI: Acute renal injury
HRs: Hazard ratio
