# Supplementary material for: Risk factors for developing ventilator-associated lower respiratory tract infection in patients with severe COVID-19: a multinational, multicentre study, prospective, observational study
Source: Sci Rep. 2023 Apr 21;13:6553. doi: 10.1038/s41598-023-32265-5 (PMC10119842; doi:10.1038/s41598-023-32265-5)
Supplement: Supplementary file 1 — Supplementary Information. [file 41598_2023_32265_MOESM1_ESM.docx]

**TITLE:** Risk Factors for Developing Ventilator-Associated Lower Respiratory Tract Infection in Patients with Severe COVID-19: A Multinational, Multicentre Study, Prospective, Observational Study

Statistical Analyisis

Categorical variables are presented in counts (percentages) and were evaluated through the Chi-square test. As appropriate, continuous variables were expressed as median (interquartile ranges) or mean (standard deviation). For continuous variables with normal distribution, the t Student test was performed, and for variables with no normal distribution Wilcoxon-Mann-Whitney test was used.

A random forest (RF) model was used to predict the probability of ICU mortality. The RF model is a type of ensemble-learning model that uses multiple decision trees as its base models, and a majority voting system is used as the final aggregation method to synthesize the classification results of all the base models. A total of 500 estimators were used in this model. To calculate the area under the model's receiver operating curve (AUROC), cross-validation was performed, which is a validation technique to evaluate how the results of a statistical analysis will generalize to an independent data set. The data set was divided into ten subsets, and the validation was repeated ten times. Each time, one of the subsets was used as the test cohort, and the other nine subsets were put together to form the training cohort. The average AUROC was then calculated across all ten trials.

Then, a recursive elimination feature was carried out to select the smallest possible subset of variables that generate a model with adequate performance based on its AUROC. This technique works by looking for a subset of characteristics starting with all the variables in the training data set and sequentially removing variables until the difference between the average AUROC of the model trained with all the variables and the model trained with the subset of variables is equal to 0.1. The elimination is done according to the Gini importance, removing the variable with the least important from the dataset. Subsequently, to interpret the contribution of the optimal subset of variables to the model, the Python Treeinterpreter library was used. The library contains functions that allow decomposing each prediction into bias and feature contribution components, as follows: *prediction = bias + feature_1_contribution + ... + feature_n_contribution.* The random forest model, which is considered a "black box," can be interpreted with this tool. We can know how the value of each variable has consequences on the outcome (*i.e.,* ICU mortality). A multivariate logistic regression model was carried out with sociodemographic, admission data, and clinical outcomes variables identified as relevant in the final RF model (independent variables) to determine the Odds ratios (ORs) related to ICU mortality.

Finally, another multivariate logistic regression model was developed to quantify the adjusted risk of VA-LRTI(outcome variable) with all the sociodemographic, admission data, and clinical outcomes variables (independent variables). Variables were selected to adjust the model with the best performance (*P* value < 0.05 in Wald tests). ORs were calculated based on the exponentials of coefficients obtained and presented in a Forest plot. A significance level of 0.05 and a confidence level of 95% were chosen. All statistical analysis was carried out in the R studio 1.3.1056, Python 3.9.5 - 3.7.9, and IBM SPSS 28 for MAC.

|  | **Latin America** | | | **Europe** | | |
| --- | --- | --- | --- | --- | --- | --- |
|  | VA-LRTI  (N= 378) | No VA-LRTI  (N= 743) | *P*-value | VA-LRTI  (N= 596) | No VA-LRTI  (N=1,906) | *P*-value |
| Age, median (IQR) | 61.0 (53.0-69.0) | 61.0 (51.0-70.0) | 0.61 | 66.0 (59.0-73.0) | 64.0 (55.0-71.0) | <0.001 |
| Male, N (%) | 258 (68.3) | 506 (68.1) | 0.99 | 439 (73.7) | 1352 (70.9) | 0.22 |
| Health worker, N (%) | 9 (2.4) | 11 (1.5) | 0.40 | 14 (2.4) | 52 (2.7) | 0.72 |
| Influenza vaccine, N (%) | 31 (8.2) | 34 (4.6) | 0.02 | 0 (0.0) | 0 (0.0) | 1.00 |
| **Comorbid condition, N (%)** | | | | | | |
| Congestive heart failure | 29 (7.7) | 93 (12.5) | 0.02 | 20 (3.4) | 56 (2.9) | 0.70 |
| Hypertension | 168 (44.4) | 347 (46.7) | 0.51 | 303 (50.8) | 867 (45.5) | 0.03 |
| COPD | 26 (6.9) | 89 (12.0) | 0.01 | 49 (8.2) | 131 (6.9) | 0.31 |
| Asthma | 2 (0.5) | 3 (0.4) | 0.86 | 38 (6.4) | 107 (5.6) | 0.55 |
| Chronic kidney disease | 36 (9.5) | 51 (6.9) | 0.15 | 25 (4.2) | 98 (5.1) | 0.41 |
| Neurologic disease | 9 (2.4) | 23 (3.1) | 0.62 | 5 (0.8) | 15 (0.8) | 0.89 |
| Haematological disease | 6 (1.6) | 9 (1.2) | 0.81 | 29 (4.9) | 55 (2.9) | 0.03 |
| HIV-AIDS | 3 (0.8) | 2 (0.3) | 0.44 | 3 (0.5) | 2 (0.1) | 0.17 |
| Obesity | 105 (27.8) | 304 (40.9) | <0.001 | 208 (34.9) | 983 (35.8) | 0.71 |
| Rheumatological disease | 4 (1.1) | 15 (2.0) | 0.35 | 18 (3.0) | 73 (3.8) | 0.43 |
| Diabetes | 107 (28.3) | 206 (27.7) | 0.89 | 149 (25.0) | 441 (23.1) | 0.38 |
| Number of comorbidities, median (IQR) | 1.0 (0.0-2.0) | 1.0 (1.0-3.0) | 0.02 | 1.0 (1.0-2.0) | 1.0 (0.0-2.0) | 0.02 |
| **Laboratory Testing, median (IQR)** | | | | | | |
| Creatinine | 0.9 (0.7-1.3) | 0.9 (0.7-1.3) | 0.11 | 0.9 (0.7-1.2) | 0.9 (0.7-1.1) | 0.66 |
| Leucocytes | 11.2 (8.1-16.1) | 10.1 (6.9-13.9) | <0.001 | 9.0 (6.5-12.8) | 8.7 (6.2-12.3) | 0.19 |
| CRP | 31.5 (13.0-174.5) | 21.0 (10.0-40.0) | <0.001 | 14.9 (8.0-23.1) | 15.2 (8.1-24.6) | 0.44 |
| PaO2 | 67.0 (56.3-83.0) | 68.0 (57.0-81.0) | 0.92 | 68.0 (58.0-81.0) | 69.0 (58.3-83.0) | 0.42 |
| FiO2 | 75.0 (50.0-100.0) | 60.0 (40.0-90.0) | <0.001 | 80.0 (50.0-100.0) | 70.0 (45.0-95.0) | <0.001 |
| PaO2/FiO2 | 99.3 (68.8-147.8) | 120.0 (79.5-190.0) | <0.001 | 96.7 (72.4-140.0) | 110.0 (77.0-173.8) | <0.001 |
| pH | 7.3 (7.3-7.4) | 7.3 (7.3-7.4) | 0.06 | 7.4 (7.3-7.4) | 7.4 (7.3-7.4) | 0.61 |
| SO2 | 92.0 (88.0-94.0) | 92.0 (88.0-93.0) | 0.03 | 92.0 (92.0-92.0) | 92.0 (92.0-92.0) | 0.63 |
| Procalcitonin | 0.3 (0.3-0.8) | 0.3 (0.3-0.3) | <0.001 | 0.3 (0.1-0.5) | 0.3 (0.1-0.1) | 0.02 |
| **Treatments and Interventions** | | | | | | |
| Hospital LOS, median (IQR) | 23.0 (15.0-39.8) | 18.0 (11.0-29.0) | <0.001 | 42.0 (28.0-58.0) | 25.0 (16.0-39.0) | <0.001 |
| ICU LOS, median (IQR) | 17.0 (12.0-27.0) | 11.0 (7.0-18.0) | <0.001 | 30.0 (20.0-46.0) | 14.0 (9.0-24.0) | <0.001 |
| IMV Days, median (IQR) | 15.0 (10.0-23.8) | 9.0 (5.0-14.0) | <0.001 | 26.0 (16.0-39.3) | 11.0 (7.0-20.0) | <0.001 |
| ECMO, N (%) | 10 (2.7) | 18 (2.4) | 0.98 | 18 (3.0) | 50 (2.6) | 0.71 |
| Prone position, N (%) | 310 (82.0) | 536 (72.1) | <0.001 | 495 (83.1) | 1,288 (67.6) | <0.001 |
| Corticosteroids use, N (%) | 284 (75.1) | 613 (82.5) | <0.01 | 444 (74.5) | 1,240 (65.1) | <0.001 |
| One Corticosteroid, N (%) | 275 (72.8) | 604 (81.3) | <0.001 | 436 (73.2) | 1,182 (62.0) | <0.001 |
| Two Corticosteroid, N (%) | 63 (16.7) | 20 (2.7) | <0.001 | 5 (0.8) | 7 (0.3) | <0.001 |
| Hydrocortisone, N (%) | 79 (20.9) | 28 (3.8) | <0.001 | 26 (4.4) | 76 (4.0) | 0.78 |
| Methylprednisolone, N (%) | 101 (26.7) | 260 (35.0) | 0.01 | 204 (34.2) | 582 (30.5) | 0.10 |
| Dexamethasone, N (%) | 221 (58.5) | 556 (47.9) | 0.001 | 216 (36.2) | 536 (28.1) | <0.001 |
| Dexamethasone+ Hydrocortisone, N (%) | 53 (14.0) | 8 (1.1) | <0.001 | 2 (0.3) | 0 (0.0) | 0.09 |
| Dexamethasone+ Methylprednisolone, N (%) | 10 (2.7) | 12 (1.6) | 0.34 | 0 (0.0) | 0 (0.0) | 1.00 |
| Methylprednisolone+ Hydrocortisone, N (%) | 0 (0.0) | 0 (0.0) | 1.00 | 3 (0.5) | 6 (0.3) | 0.78 |
| **Outcomes** | | | | | | |
| AKI, N (%) | 170 (45.0) | 304 (40.9) | 0.22 | 237 (39.8) | 555 (29.1) | <0.001 |
| Deaths, N (%) | 190 (50.3) | 320 (43.1) | 0.03 | 246 (41.3) | 684 (35.9) | 0.02 |

INTENTO 1

|  | **All Cohort**  **(N= 3,287)** | **VAP**  **(N= 610)** | **No VAP**  **(N= 2,677)** | ***P*-value VAP** | **VAT**  **(N= 338)** | **No VAT**  **(N= 2,949)** | ***P*-value  VAT** |
| --- | --- | --- | --- | --- | --- | --- | --- |
| Age, median (IQR) | 63.0 (54.5-71.0) | 65.0 (57.0-72.0) | 63.0 (54.0-71.0) | <0.001 | 62.0 (54.0-69.8) | 64.0 (55.0-71.0) | 0.09 |
| Male, N (%) | 2,317 (70.5) | 442 (72.5) | 1,875 (70.0) | 0.26 | 236 (69.8) | 2,081 (70.6) | 0.83 |
| Health worker, N (%) | 79 (2.4) | 12 (2.0) | 67 (2.5) | 0.53 | 9 (2.7) | 70 (2.4) | 0.89 |
| Influenza vaccine, N (%) | 59 (1.8) | 1 (0.2) | 58 (2.2) | 0.001 | 29 (8.6) | 30 (1.0) | <0.001 |
| First Wave | 2,516 (76.5) | 371 (60.8) | 21.5 (80.1) | <0.001 | 302 (89.4) | 2214 (75.1) | <0.001 |
| Second Wave, N(%) | 771 (23.5) | 239 (39.2) | 532 (19.9) | <0.001 | 36 (10.6) | 735 (24.9) | <0.001 |
| **Comorbid condition, N (%)** | | | | | | | |
| Congestive heart failure | 172 (5.2) | 22 (3.6) | 150 (5.6) | 0.06 | 23 (6.8) | 149 (5.1) | 0.21 |
| Hypertension | 1,532 (46.6) | 311 (51.0) | 1,221 (45.6) | 0.018 | 145(42.9) | 1387 (47.0) | 0.17 |
| COPD | 250 (7.6) | 51 (8.4) | 199 (7.4) | 0.49 | 22 (6.5) | 228 (7.7) | 0.49 |
| Asthma | 140 (4.3) | 33 (5.4) | 107 (4.0) | 0.15 | 4(1.2) | 136 (4.6) | 0.004 |
| Chronic kidney disease | 185 (5.6) | 26 (4.3) | 159 (5.9) | 0.13 | 30 (8.9) | 155 (5.3) | 0.009 |
| Neurologic disease | 47 (1.4) | 7 (1.2) | 40 (1.5) | 0.64 | 7 (2.1) | 40 (1.4) | 0.42 |
| Haematological disease | 90 (2.7) | 29 (4.8) | 61 (2.3) | 0.001 | 5 (1.5) | 85 (2.9) | 0.19 |
| HIV-AIDS | 10 (0.3) | 2 (0.3) | 8 (0.3) | 0.77 | 4 (1.2) | 6 (0.2) | 0.010 |
| Obesity | 1,1960 (36.4) | 221 (36.2) | 975 (36.4) | 0.97 | 85 (25.2) | 1,111 (37.7) | <0.001 |
| Rheumatological disease | 105 (3.2) | 17 (2.8) | 88 (3.3) | 0.61 | 4 (1.2) | 101 (3.4) | 0.039 |
| Diabetes | 819 (24.9) | 156 (25.6) | 662 (24.7) | 0.70 | 93 (27.5.3) | 725 (24.6) | 0.27 |
| ARDS at admission | 721 (2.9) | 59 (9.7) | 662 (24.7) | <0.001 | 183 (54.1) | 538 (18.2) | <0.001 |
| Number of comorbidities, median (IQR) | 1.0 (0.0-2.0) | 1.0 (1.0-2.0) | 1.0 (0.0-2.0) | 0.06 | 1.0 (0.0-2.0) | 1.0 (0.0-2.0) | 0.028 |
| **Laboratory Testing, median (IQR)** | | | | | | | |
| Creatinine | 0.9 (0.7-1.2) | 0.9 (0.7-1.2) | 0.9 (0.7-1.2) | 0.72 | 0.9 (0.7-1.2) | 0.9 (0.7-1.2) | 0.60 |
| Leucocytes | 9.3 (6.6-13.1) | 9.4 (6.7-13.2) | 9.3 (6.6-13.1) | 0.66 | 11.1 (7.8-15.8) | 9.1 (6.5-12.9) | <0.001 |
| CRP | 16.7 (9.0-28.5) | 15.6 (97.8-25.0) | 17.0 (9.1-29.3) | <0.001 | 27.2 (12.0-152.5) | 16.0 (8.7-27.0) | <0.001 |
| Procalcitonin | 0.3 (0.2-0.4) | 0.3 (0.1-0.4) | 0.3 (0.2-0.4) | 0.003 | 0.3 (0.3-0.8) | 0.3 (0.2-0.4) | <0.001 |

| **Treatments and Interventions** | | | | | | | |
| --- | --- | --- | --- | --- | --- | --- | --- |
|  | **All Cohort**  **(N= 3,287)** | **VAP**  **(N= 610)** | **No VAP**  **(N= 2,677)** | ***P*-value VAP** | **VAT**  **(N= 338)** | **No VAT**  **(N= 2,949)** | ***P*-value  VAT** |
| Hospital LOS, median (IQR) | 26.0 (17.0-42.5) | 40.0 (27.0-57.0) | 24.0 (15.0-18.0) | <0.001 | 24.5 (16.0-44.0) | 27.0 (17.0-42.0) | 0.66 |
| ICU LOS, median (IQR) | 17.0 (10.0-28.0) | 29.0 (19.0-45.0) | 15.0 (9.9 -23.0) | < 0.001 | 18.0 (12.0-28.0) | 16.0 (10.0-28.0) | 0.007 |
| IMV Days, median (IQR) | 14.0 (8.0-24.0) | 25.0 (16.0-38.0) | 12.0 (7.0-20.0) | <0.001 | 15.0 (11.0-25.0) | 14.0 (8.0-24.0) | 0.004 |
| ECMO, N (%) | 92 (2.8) | 21 (3.4) | 71 (2.6) | 0.35 | 7 (2.1) | 85 (2.9) | 0.49 |
| Prone position, N (%) | 2,498 (76.0) | 507 (83.1) | 1,991 (74.4) | <0.001 | 277 (82.0) | 2,221 (75.3) | 0.008 |
| Corticosteroids use, N (%) | 2,413 (73.4) | 460 (75.4) | 1,953 (73.0) | 0.23 | 249 (73.7) | 2,164 (73.4) | 0.96 |
| One Corticosteroid, N (%) | 2,363 (71.9) | 457 (74.9) | 1,906 (71.2) | 0.18 | 238 (70.4) | 2,125 (72.1) | <0.001 |
| Two Corticosteroid, N (%) | 92 (2.8) | 16 (2.6) | 76 (2.8) | 0.18 | 50 (14.8) | 42 (1.4) | <0.001 |
| Hydrocortisone, N (%) | 200 (6.1) | 36 (5.9) | 164 (6.1) | 0.91 | 66 (19.5) | 134 (4.5) | <0.001 |
| Methylprednisolone, N (%) | 1,096 (31.4) | 200 (32.8) | 896(33.5) | 0.78 | 98 (29.0) | 998 (33.8) | 0.08 |
| Dexamethasone, N (%) | 1,251 (38.1) | 2,530 (41.5) | 998 (37.3) | 0.06 | 174 (51.5) | 1, 077 (36.5) | <0.001 |
| Dexamethasone+ Hydrocortisone, N (%) | 61 (1.9) | 12 (2.0) | 49 (1-8) | 0.95 | 41 (12.1) | 20 (0.7) | <0.001 |
| Dexamethasone+ Methylprednisolone, N (%) | 22 (0.7) | 1 (0.2) | 21 (0.8) | 0.16 | 9 (2.7) | 13 (0.4) | <0.001 |
| Methylprednisolone+ Hydrocortisone, N (%) | 9 (0.3) | 3 (0.5) | 6 (0.2) | 0.48 | 0 (0.0) | 9 (0.3) | 0.64 |
| **Outcomes** | | | | | | | |
| AKI, N (%) | 1,147 (34.9) | 256 (42.0) | 891(33.3) | <0.001 | 140 (41.4) | 1,007 (34.2) | 0.027 |
| Deaths, N (%) | 1,285 (39.1) | 268 (43.9) | 1,017 (38.0) | 0.007 | 160 (47.3) | 1,125 (38.2) | 0.001 |

INTENTO 2

|  | **All Cohort**  **(N= 3,287)** | **VAP**  **(N= 610)** | **No VAP**  **(N= 2,677)** | ***P*-value VAP** | **VAT**  **(N= 338)** | **No VAT**  **(N= 2,949)** | ***P*-value  VAT** |
| --- | --- | --- | --- | --- | --- | --- | --- |
| Age, median (IQR) | 63.0 (54.5-71.0) | 65.0 (57.0-72.0) | 63.0 (54.0-71.0) | <0.001 | 62.0 (54.0-69.8) | 64.0 (55.0-71.0) | 0.09 |
| Male, N (%) | 2,317 (70.5) | 442 (72.5) | 1,875 (70.0) | 0.26 | 236 (69.8) | 2,081 (70.6) | 0.83 |
| Health worker, N (%) | 79 (2.4) | 12 (2.0) | 67 (2.5) | 0.53 | 9 (2.7) | 70 (2.4) | 0.89 |
| Influenza vaccine, N (%) | 59 (1.8) | 1 (0.2) | 58 (2.2) | 0.001 | 29 (8.6) | 30 (1.0) | <0.001 |
| First Wave | 2,516 (76.5) | 371 (60.8) | 21.5 (80.1) | <0.001 | 302 (89.4) | 2214 (75.1) | <0.001 |
| Second Wave, N(%) | 771 (23.5) | 239 (39.2) | 532 (19.9) | <0.001 | 36 (10.6) | 735 (24.9) | <0.001 |
| **Comorbid condition, N (%)** | | | | | | | |
| Congestive heart failure | 172 (5.2) | 22 (3.6) | 150 (5.6) | 0.06 | 23 (6.8) | 149 (5.1) | 0.21 |
| Hypertension | 1,532 (46.6) | 311 (51.0) | 1,221 (45.6) | 0.018 | 145(42.9) | 1387 (47.0) | 0.17 |
| COPD | 250 (7.6) | 51 (8.4) | 199 (7.4) | 0.49 | 22 (6.5) | 228 (7.7) | 0.49 |
| Asthma | 140 (4.3) | 33 (5.4) | 107 (4.0) | 0.15 | 4(1.2) | 136 (4.6) | 0.004 |
| Chronic kidney disease | 185 (5.6) | 26 (4.3) | 159 (5.9) | 0.13 | 30 (8.9) | 155 (5.3) | 0.009 |
| Neurologic disease | 47 (1.4) | 7 (1.2) | 40 (1.5) | 0.64 | 7 (2.1) | 40 (1.4) | 0.42 |
| Haematological disease | 90 (2.7) | 29 (4.8) | 61 (2.3) | 0.001 | 5 (1.5) | 85 (2.9) | 0.19 |
| HIV-AIDS | 10 (0.3) | 2 (0.3) | 8 (0.3) | 0.77 | 4 (1.2) | 6 (0.2) | 0.010 |
| Obesity | 1,1960 (36.4) | 221 (36.2) | 975 (36.4) | 0.97 | 85 (25.2) | 1,111 (37.7) | <0.001 |
| Rheumatological disease | 105 (3.2) | 17 (2.8) | 88 (3.3) | 0.61 | 4 (1.2) | 101 (3.4) | 0.039 |
| Diabetes | 819 (24.9) | 156 (25.6) | 662 (24.7) | 0.70 | 93 (27.5.3) | 725 (24.6) | 0.27 |
| ARDS at admission | 721 (2.9) | 59 (9.7) | 662 (24.7) | <0.001 | 183 (54.1) | 538 (18.2) | <0.001 |
| Number of comorbidities, median (IQR) | 1.0 (0.0-2.0) | 1.0 (1.0-2.0) | 1.0 (0.0-2.0) | 0.06 | 1.0 (0.0-2.0) | 1.0 (0.0-2.0) | 0.028 |
| **Laboratory Testing, median (IQR)** | | | | | | | |
| Creatinine | 0.9 (0.7-1.2) | 0.9 (0.7-1.2) | 0.9 (0.7-1.2) | 0.72 | 0.9 (0.7-1.2) | 0.9 (0.7-1.2) | 0.60 |
| Leucocytes | 9.3 (6.6-13.1) | 9.4 (6.7-13.2) | 9.3 (6.6-13.1) | 0.66 | 11.1 (7.8-15.8) | 9.1 (6.5-12.9) | <0.001 |
| CRP | 16.7 (9.0-28.5) | 15.6 (97.8-25.0) | 17.0 (9.1-29.3) | <0.001 | 27.2 (12.0-152.5) | 16.0 (8.7-27.0) | <0.001 |
| Procalcitonin | 0.3 (0.2-0.4) | 0.3 (0.1-0.4) | 0.3 (0.2-0.4) | 0.003 | 0.3 (0.3-0.8) | 0.3 (0.2-0.4) | <0.001 |

| **Treatments and Interventions** | | | | | | | |
| --- | --- | --- | --- | --- | --- | --- | --- |
|  | **All Cohort**  **(N= 3,287)** | **VAP**  **(N= 610)** | **No VAP**  **(N= 2,677)** | ***P*-value VAP** | **VAT**  **(N= 338)** | **No VAT**  **(N= 2,949)** | ***P*-value  VAT** |
| Hospital LOS, median (IQR) | 26.0 (17.0-42.5) | 40.0 (27.0-57.0) | 24.0 (15.0-18.0) | <0.001 | 24.5 (16.0-44.0) | 27.0 (17.0-42.0) | 0.66 |
| ICU LOS, median (IQR) | 17.0 (10.0-28.0) | 29.0 (19.0-45.0) | 15.0 (9.9 -23.0) | < 0.001 | 18.0 (12.0-28.0) | 16.0 (10.0-28.0) | 0.007 |
| IMV Days, median (IQR) | 14.0 (8.0-24.0) | 25.0 (16.0-38.0) | 12.0 (7.0-20.0) | <0.001 | 15.0 (11.0-25.0) | 14.0 (8.0-24.0) | 0.004 |
| ECMO, N (%) | 92 (2.8) | 21 (3.4) | 71 (2.6) | 0.35 | 7 (2.1) | 85 (2.9) | 0.49 |
| Prone position, N (%) | 2,498 (76.0) | 507 (83.1) | 1,991 (74.4) | <0.001 | 277 (82.0) | 2,221 (75.3) | 0.008 |
| Corticosteroids use, N (%) | 2,413 (73.4) | 460 (75.4) | 1,953 (73.0) | 0.23 | 249 (73.7) | 2,164 (73.4) | 0.96 |
| One Corticosteroid, N (%) | 2,363 (71.9) | 457 (74.9) | 1,906 (71.2) | 0.18 | 238 (70.4) | 2,125 (72.1) | <0.001 |
| Two Corticosteroid, N (%) | 92 (2.8) | 16 (2.6) | 76 (2.8) | 0.18 | 50 (14.8) | 42 (1.4) | <0.001 |
| Hydrocortisone, N (%) | 200 (6.1) | 36 (5.9) | 164 (6.1) | 0.91 | 66 (19.5) | 134 (4.5) | <0.001 |
| Methylprednisolone, N (%) | 1,096 (31.4) | 200 (32.8) | 896(33.5) | 0.78 | 98 (29.0) | 998 (33.8) | 0.08 |
| Dexamethasone, N (%) | 1,251 (38.1) | 2,530 (41.5) | 998 (37.3) | 0.06 | 174 (51.5) | 1, 077 (36.5) | <0.001 |
| Dexamethasone+ Hydrocortisone, N (%) | 61 (1.9) | 12 (2.0) | 49 (1-8) | 0.95 | 41 (12.1) | 20 (0.7) | <0.001 |
| Dexamethasone+ Methylprednisolone, N (%) | 22 (0.7) | 1 (0.2) | 21 (0.8) | 0.16 | 9 (2.7) | 13 (0.4) | <0.001 |
| Methylprednisolone+ Hydrocortisone, N (%) | 9 (0.3) | 3 (0.5) | 6 (0.2) | 0.48 | 0 (0.0) | 9 (0.3) | 0.64 |
| **Outcomes** | | | | | | | |
| AKI, N (%) | 1,147 (34.9) | 256 (42.0) | 891(33.3) | <0.001 | 140 (41.4) | 1,007 (34.2) | 0.027 |
| Deaths, N (%) | 1,285 (39.1) | 268 (43.9) | 1,017 (38.0) | 0.007 | 160 (47.3) | 1,125 (38.2) | 0.001 |
